# Acute hemodynamic effect of inhaled iloprost in pulmonary artery hypertension evaluated with echocardiography

**DOI:** 10.1186/1476-7120-5-41

**Published:** 2007-11-21

**Authors:** Maria José Loureiro, Carlos Cotrim, Otília Simões, Rita Miranda, Pedro Cordeiro, Manuel Carrageta

**Affiliations:** 1 Cardiology Department, Garcia de Orta Hospital, Almada, Portugal

## Abstract

Doppler echocardiography is useful in the initial evaluation and long-term follow-up of patients with pulmonary artery hypertension. Aerosolised iloprost has been shown to reduce pulmonary pressure immediately after inhalation. We report the echocardiographic findings in a patient with severe pulmonary hypertension, before and after the inhalation of aerosolized iloprost. These findings illustrate the acute influence of iloprost in right and left ventricular hemodynamics and morphology. These findings were reproduced in subsequent echocardiographic evaluations.

## Introduction

Doppler echocardiography is useful in the initial evaluation and long-term follow-up of patients with pulmonary arterial hypertension [1,2].

## Case presentation

**Figure 1:**
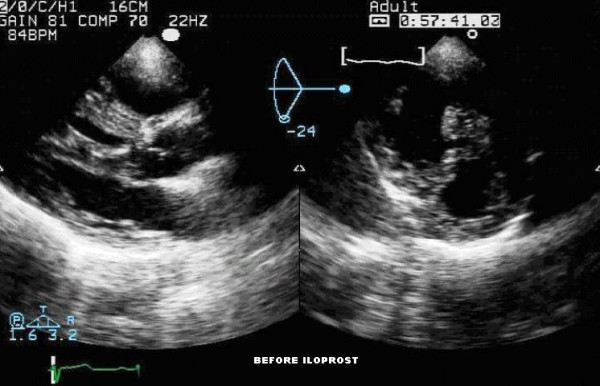
Transthoracic echocardiogram showing left ventricle long and short axis views before iloprost nebulization. This echocardiography reveals the severe right ventricular dilatation and hypertrophy. As the right ventricle undergoes dilation and hypertrophy, its crescentic shape is lost and the septum shifts leftward compressing the left ventricle. This septum shift leads to a proportional reduction in left ventricular dimension and impaired left ventricular systolic performance.

**Figure 2:**
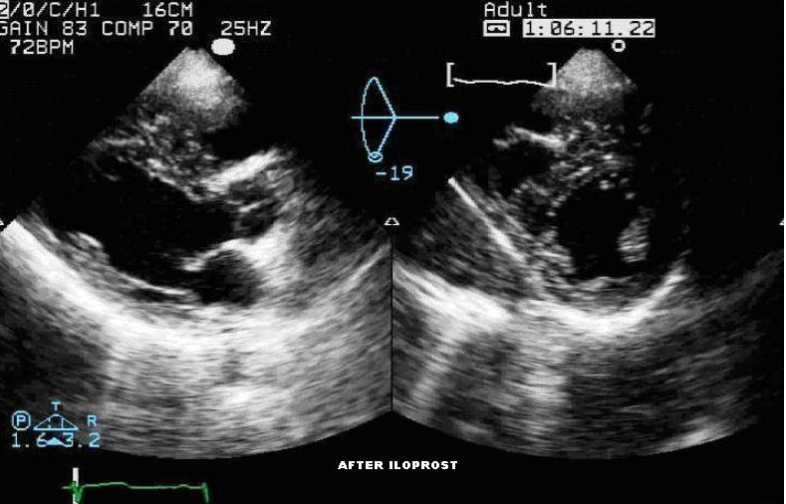
Transthoracic echocardiogram showing left ventricle long and short axis views after iloprost nebulization. In the echocardiographic evaluation after iloprost administration we can detect a reduction in right ventricular dimension, with a proportional increase in left ventricular dimension and performance. The correction of the septal shift secondary to right ventricular overload also contributes to a smaller right ventricle and expanded left ventricle.

**Figure 3:**
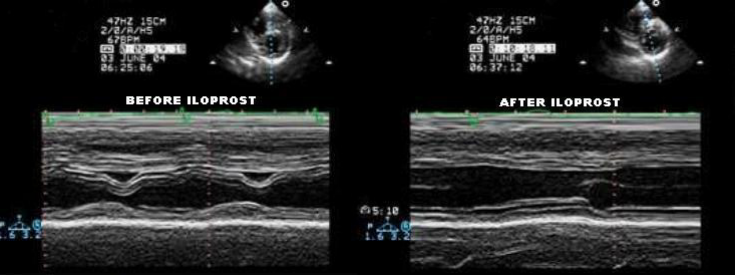
M-mode tracing at the left ventricular level before (left) and after (right) iloprost nebulization. On the left hand side this short axis M-mode tracing shows the diastolic intraventricular septal shifting toward the left ventricle, leading to its marked underfilling. On the right hand side of the figure we can observe the correction of the septal shift after iloprost nebulization.

A 21-year-old woman diagnosed with idiopathic pulmonary hypertension since she was eleven, responsive to calcium channel blockers, was referred for Cardiology consultation at 23 weeks pregnancy. She was on nifedipine and fraxiparine and Doppler echocardiography revealed a dilated and hypertrophied right ventricle and a peak tricuspid regurgitant jet velocity of 5.3 ms^-1 ^with estimated right ventricular systolic pressure (RVSP) of 122 mmHg. She was then started on 5.0 μg of nebulized iloprost six times per day. At the 29^th ^week of pregnancy Doppler echocardiography was repeated before and immediately after a single dose of 5 μg of iloprost. Postinhalation acute assessment revealed not only a remarkable reduction in RVSP but also an impressive change in cardiac morphology as is evident from figures 1, 2 and 3 and from two-dimensional and three-dimensional echocardiography video clips (see Additional files 1, 2, 3 and 4). These findings were reproduced in subsequent echocardiographic evaluations and illustrate the acute hemodynamic effects of iloprost nebulization in a patient with severe pulmonary hypertension.

## Conclusion

The findings that we describe showed the potential utility of echocardiography in the evaluation of the acute effects of drugs used in the treatment of pulmonary arterial hypertension.

## Supplementary Material

Additional file 1 Two-dimensional parasternal view before iloprost inhalation. Two-dimensional parasternal long and short axis views before iloprost inhalation showing a small left ventricle with increased eccentricity index. Click here for file

Additional file 2 Two-dimensional parasternal view after iloprost inhalation. Two-dimensional parasternal long and short axis views after iloprost inhalation showing near normal morphology of both ventricles. Click here for file

Additional file 3 Three-dimensional parasternal view before iloprost inhalation. Three-dimensional parasternal long axis view before iloprost inhalation showing a small left ventricle with increased eccentricity index. Click here for file

Additional file 4 Three-dimensional parasternal view after iloprost inhalation. Three-dimensional parasternal long axis view after iloprost inhalation showing near normal morphology of both ventricles. Click here for file

## References

[B1] Bossone E, Bodini B, Mazza A, Allegra L (2005). Pulmonary arterial hypertension. The key role of echocardiography. Chest.

[B2] Burgess M, Bright-Thomas R, Ray S (2002). Echocardiographic evaluation of right ventricular function. Eur J Echocardiography.

